# Histologic and histomorphometric evaluation of Aloe vera adjunctive to β-tricalcium phosphate in class II furcation defects in dogs

**DOI:** 10.1038/s41598-023-31282-8

**Published:** 2023-03-14

**Authors:** Maha R. Taalab, Samia S. Abd El Rehim, Dina W. Eldeeb, Riham M. El-Moslemany, Hams Abdelrahman

**Affiliations:** 1 Oral Medicine, Periodontology, Oral Diagnosis and Radiology Department, Faculty of Dentistry, Alexandria University, Alexandria, Egypt; 2 Oral Biology Department, Faculty of Dentistry, Alexandria University, Alexandria, Egypt; 3 Pharmaceutics Department, Faculty of Pharmacy, Alexandria University, Alexandria, Egypt; 4 Dental Public Health Department, Faculty of Dentistry, Alexandria University, Alexandria, Egypt

**Keywords:** Health care, Medical research

## Abstract

Aloe vera has antimicrobial activity and enhances the osseointegration process, thus it may have the potential in treating periodontal defects. This study aimed to evaluate the effect of Aloe vera as an adjunction to Beta-tricalcium phosphate (β-TCP) bone graft in Grade II furcation defects. A randomized study was conducted on six healthy mongrel dogs' premolars. A total of twenty-four Grade II furcation critical-sized defects were surgically created after reflecting a full-thickness flap, twelve defects were filled with β-TCP while the other twelve defects were filled with Aloe vera mixed with β-TCP and both covered by collagen membrane. Animals were euthanized at the end of the fourth and eighth week and defects were analyzed histologically and histomorphometrically. Histologically, Aloe vera mixed with β-TCP resulted in more bone formation and new PDL fibers compared to β-TCP alone. After 2 and 4 weeks, the experimental group had significantly higher newly formed interradicular bone height (*p* < 0.0001, and *p* < 0.0001, respectively), bone thickness (*p* < 0.0001, and *p* < 0.0001, respectively), and percentage of the surface area (*p* = 0.009, and *p* = 0.023, respectively). Aloe vera gel adjunctive to β-TCP is an effective bioactive agent that enhances periodontal tissue regeneration and bone formation in critically sized defects.

## Introduction

Periodontitis affects multi-rooted teeth, resulting in furcation involvement, and leading to a higher rate of tooth loss than those without furcation defects^1^. The main clinical challenge for the treatment of furcation defects is the complexity of their anatomy which impairs accessibility for debridement^2,3^. Thus, surgical treatment using guided tissue regeneration (GTR) was used to regenerate bone in Grade II furcation involvement as it showed superior results than open debridement^4^.

GTR using either resorbable or non-resorbable membranes over different bone grafts has been used for the treatment of furcation defects in humans^5,6^. Different biological agents such as enamel matrix derivative, platelet-derived growth factor, platelet-rich plasma, or platelet-rich plasma fibrin proved to be successful in periodontal regeneration in Grade II furcation defects^7^.

Recently, Agrawal et al.^8^ reported that Bone Morphogenic Protein 2 (rhBMP-2) adjunctive to (PLA/PGA) demonstrated improvement in the periodontal tissue regeneration of Grade II furcation defects resulting in accelerated bone formation without any aberrant healing events as root resorption and ankylosis complicating the regenerative procedure. Although the gain of clinical attachment level (CAL) and bone fill are commonly used as clinical indicators for successful regeneration, only histological analysis can provide evidence for the type of newly formed tissue and the amount of regeneration achieved by GTR^9^.

Herbal medicine depends on roots, stems, leaves, flowers, or seeds of plants to improve health, prevent disease, and treat illness. Plant based conventional drugs were proven to be effective, safe, and more affordable^10^.

Aloe vera is a cactus plant that belongs to the Liliaceae family. It contains about 75 potentially active constituents such as vitamins, enzymes, minerals, sugars, lignin, saponins, salicylic acids, amino acids, and polysaccharides. It has an anti-inflammatory effect with immunoregulatory properties thus it has drawn attention to be used in the medical field^11^. It has been tested for the treatment of damaged tissues and has shown therapeutic properties as being anti-inflammatory, antibacterial, hypoglycemic, immunomodulator, wound healer, and promotor for tissue regeneration^12^.

The antimicrobial properties of Aloe vera have been demonstrated to be successful in combating gram-negative bacteria, which are the main microorganisms associated with the cause of periodontitis^13^. Additionally, Aloe vera contains a polysaccharide biomolecule called Acemannan that was reported to play a significant role in cell proliferation, and osteogenesis through stimulating extracellular matrix synthesis, mineralization, and increasing the mRNA expression of bone morphogenetic protein 2 (BMP-2). Moreover, it was reported that Acemannan can encourage the regeneration of periodontal tissues including alveolar bone, periodontal ligament, and cementum^14–16^.

Recently, it has been reported that Aloe vera can improve the osseointegration process when employed as a coating for Polylactic acid (PLA)-based scaffolds. Aloe vera has the advantage of high availability with affordable prices, which makes it a good choice from an economic point of view compared to other commercially available bioactive products^17,18^.

To clarify the regenerative and osteoinductive effect of Aloe vera that may improve the overall outcome of the GTR procedure in managing furcation bony defects, more evidence must be gathered. Therefore, this study has been proposed to assess histologically the effect of using Aloe vera on the healing of surgically created Grade II furcation defects treated by GTR in adult healthy Mongrel dogs. The null hypothesis of this study proposed that there would be no significant difference in the regeneration of Grade II furcation defects in dogs with or without using Aloe vera.

## Methods

The Ethical Committee of Alexandria University, which includes the institutional experimentation committee, approved the research protocol (IRB 00010556-IORG 0008839). This animal experiment followed the National Institutes of Health guide for the care and use of laboratory animals (National Institutes of Health Publications No. 8023, revised 1978). The study was reported according to the updated ARRIVE 2.0 guidelines. The required sample size was calculated to be 12 defects per group using statistical power software (G^*^power version 3.1.9.2; University of Düsseldorf, Germany, http://www.gpower.hhu.de/) based on 5% alpha error, 80% power, and mean difference of 0.6 [± 0.5] mm in bone height between experimental and control groups after 8 weeks which was adopted from a previous study^19^ and yielding a standardized effect size of 1.20. Six clinically healthy mongrel dogs (Canis Familiaris) between 18 and 24 months of age and weighing 20–24 kg were included. The study included twenty-four surgically induced critical-sized Grade II furcation defects in the buccal surface of the right and left mandibular premolars (P_3,_ P_4_)^19^. The twenty-four defects were randomly assigned into one of the two groups. Group I (experimental) was managed with Aloe vera gel, β-TCP allograft (Dental adbone; TCP, Medbone Biomaterials; Sintra, Portugal), and collagen membrane (Hypo Sorb; Bioimplon, Gießen, Germany)while group II (control) was managed with β-TCP and collagen membrane only.

### Aloe vera preparation

Aloe vera gel was prepared by the Department of Pharmaceutics, Faculty of Pharmacy, Alexandria University according to the updated Egyptian Guidelines for Registration of Herbal Medicines, 2021^20^. First fresh leaves of Aloe vera (Aloe barbadensis) were longitudinally sliced and the colorless gel inside was collected and homogenized at 10,000 rpm (T 25 Digital ULTRA—TURRAKX; IKA Werke, Staufen, Germany) to a liquid form. Aloe vera gel formulation was prepared by soaking 1% Carbopol 940 (Carbopol Polymer; Pharmaceuticals & Chemical industries, Alexandria, Egypt) in distilled water for 2 h. Aloe vera was then mixed with Carbopol dispersion to a final concentration of 8% w/v. After this, methylparaben (Parabens preservative; El-Gomhouria Trade Pharmaceuticals and Chemicals, Alexandria, Egypt) was added to the mixture with 0.2% dissolved in hot water and magnetically stirred (Slendor Magnetic Stirrer; Cangzhou Festar Instrument, Hebei, China) for 30 min. The pH was adjusted with 1N NaOH added dropwise with gentle stirring with a spatula until the desired pH value (6.5–7) reached^16,21^.

### Surgical procedure

The animals were anesthetized by intravenous injection of sodium thiopental (Thiopental; Sandoz, Kundl, Austria) (1:100,000) epinephrine with a dose of (13 mg/kg). A sulcular incision at the buccal aspect of the mandibular third (P_**3**_) and fourth (P_**4**_) premolars was made in the right and left quadrants. The mucoperiosteal flap was fully reflected, then a rotary 0.5 mm rounded carbide bur was used to create Grade-II critical-sized defects of 3 × 4 mm^2^ and 3.5 × 4.5 mm^2^ at furcation area in P3 and P4, respectively for each dog^19,22^. The inter-radicular bone was removed under copious irrigation using sterile saline.

**Figure 1:**
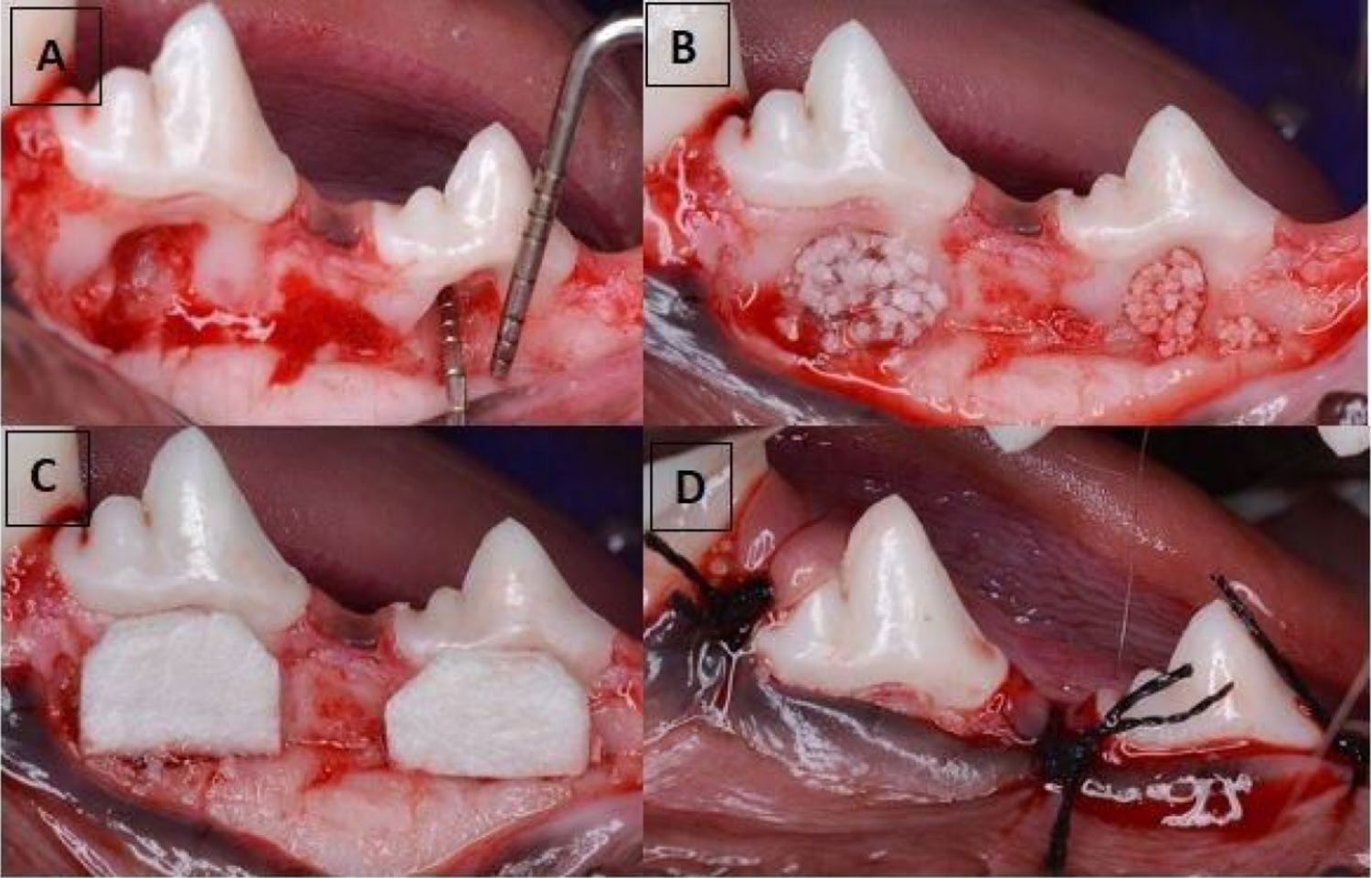
**(A)** The use of the periodontal probe to measure the vertical and horizontal dimensions of the surgically induced class II furcation defect in mandibular third (P3) and fourth (P4) premolars. (**B)** P3 defect filled with β-TCP only and P4 filled with β-TCP mixed in Aloe vera gel (**C)** Adaptation of trimmed collagen membranes over the grafted defects (**D)** Proper closure of the defects by the interproximal interrupted sutures.

Two reference points were made on the mesial and distal root surface at the bone level surrounding the created defects at the time of the surgery for future histological examination^23^. Root planing by universal curette and root conditioning using Ethylenediaminetetraacetic Acid *(*EDTA*)* (MD Chelcream; META BIOMED, Cheongiu-si, South Korea) were carried out to the exposed root surfaces. The defects of the control group were filled with β-TCP alone, while the defects of the experimental group were filled with Aloe vera gel mixed with β-TCP (1:1)^19^. The collagen membranes were then trimmed to the proper size extending 2–3 mm all around the created defects and applied over the graft filled defects. The flap was repositioned coronally to have complete coverage of the defect area and sutured with simple interrupted sutures using 3/0 silk sutures (Mersilk; Ethicon Johnson & Johnson, Somerville, NJ, USA) (Fig. 1).

### Postoperative care

Intramuscular injections of ampicillin (1 g) (Alzental; Eipico, Tenth of Ramadan City, Egypt) and Ibuprofen (600 mg) (Brufen; Abbot, Mannheim, Germany) were given to the dogs on the first day, postoperatively. The dogs received the same medication mixed with their food for one week. Throughout the study period, the dogs were fed a soft diet to minimize the possibility of local trauma at the site of the operation. Sutures were removed after 10 days.

### Clinical signs of inflammation

Every 2 weeks the dogs were anesthetized for teeth cleaning and inspecting the presence of any clinical sign of inflammation or exposure of the graft. The surgical sites were also palpated to check the presence of pus oozing from the gingival sulcus^24^.

### Animal euthanasia

Three dogs were euthanized after 4 weeks, postoperatively while the other three dogs were euthanized after 8 weeks. The euthanasia was performed using an overdose of intravenous injection of concentrated thiopental sodium (Thiopental; Sandoz, Kundl, Austria).

### Histological procedure

After euthanasia, the mandibles were dissected, and the jaw segments containing the operated teeth were separated and fixed in 10٪ neutral buffered formalin. They were decalcified and processed to obtain 5-micron mesiodistal serial sections. These sections were stained with Hematoxylin and Eosin (H&E) and Gomori trichrome then examined by light microscope to evaluate the regenerative potential in the defects and perform the morphometric analysis.

### Histomorphometry quantitative analysis

Light microscopic, photomicrographs were quantitatively analyzed at standardized magnification using Image J software (Image J version 1.46r; NIH, Bethesda, [MD], USA, https://imagej.nih.gov/ij/download.html)^25^. Three variables were analyzed: Height of newly formed bone in the interradicular region in millimeters (mm). This is the distance from the most coronal point of the formed bone till a line crossing between the two notches on the mesial and distal roots at the most apical end with a standardized magnification of 40× , the thickness of newly formed bone trabeculae in mm with a standardized magnification of 100× and the mean percentage of the newly formed bone surface area with a standardized magnification of 100×.

From each of the 24 specimens of the defects, two standardized depths were selected from which two sections were obtained. Hence a total of 48 sections were analyzed of which 24 sections (12 sections for the 4th-week group and 12 sections for the 8th-week group) belonged to the control group and the other 24 sections (12 sections for the 4th-week group and 12 sections for the 8th-week group) belonged to the experimental group. One image was taken from the chosen sections and measurements were recorded and statistically analyzed. The histological analysis was carried out by one trained investigator who was blinded to the intervention groups. Each measurement was taken 2 times, two days apart, and the mean was then recorded. (Intraexaminer correlation coefficient = 0.86, 95% confidence interval = 0.81–0.92).

### Statistical analysis

Data were tested for normality using the Shapiro–Wilk test and Q-Q plots. Normal distribution was confirmed for bone height, surface area, and thickness, therefore, the intervention in the two groups was analyzed with unpaired t-test, while the mean differences between the 4- and 8-weeks, were compared using paired t-test. Tests were two-tailed, and the significance level was set at a *p*-value of 0.05. Data were analyzed using dedicated statistical software (SPSS version 28.0; IBM, Armonk, NY, USA, https://www.ibm.com/spss).

### Ethics approval

Dogs were housed under specific pathogen-free conditions. All animal experiments were approved by the Ethics Committee of Animal Care and Experimentation of the Faculty of Dentistry, Alexandria University (IRB 00010556-IORG 0008839).

## Results

### Clinical observations

All the surgical sites showed no signs of infection or any adverse reactions after the surgery and throughout the whole study period.

### Histological findings

#### First observation period (4 weeks)

A. Experimental group

**Figure 2:**
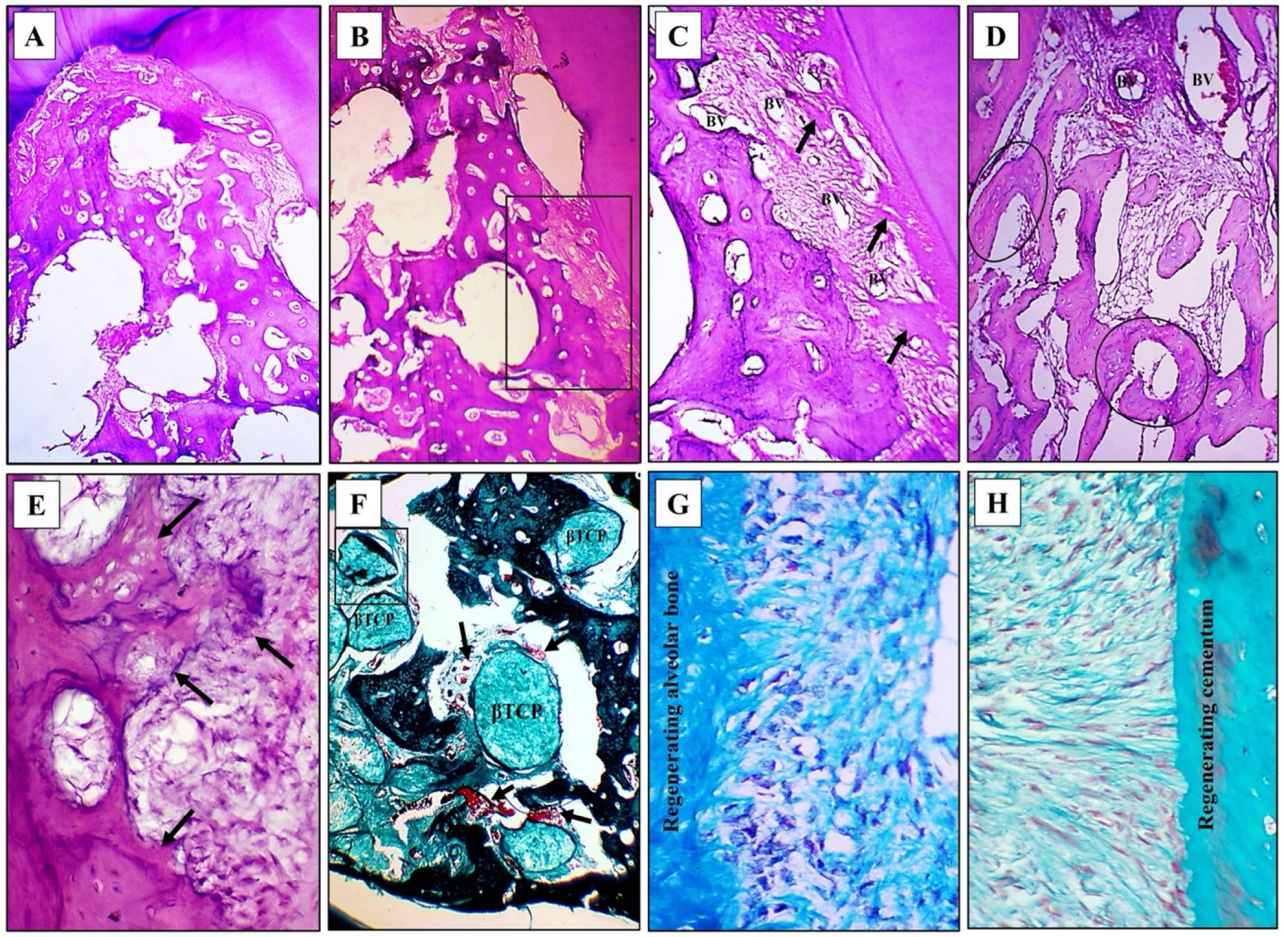
Experimental group at 4 weeks observational period **(A,B)** two successive images from one section revealing the formation of regenerative bone in wide areas of the defect with the formation of PDL fibers spanning the regions between the roots and the forming bone (insets), H&E, 40×. **(C)** Higher magnification of the two insets reveals the formation of PDL thin and irregular fibers spanning the distances between the roots and the forming bone. Note the homogeneous fiber patches (arrows) and the blood vessels among the regenerating irregular fibers (BV), H&E, 100×. **(D)** Formation of sheets of fibrous tissue between forming trabeculae and connecting them. These bone trabeculae consist partly of woven bone (circles), H&E, 100×. **(E)** Remodeling of the regenerating bone adjacent to the forming PDL (arrows), H&E, 100×. **(F)** Bone formation in close association with circular aggregates of β-TCP with accompanying rich blood supply (arrows), Trichrome stain, 100×. **(G)** Osteoblasts are arranged at the regenerating alveolar bone side and separated by the inserting fibers of the regenerating PDL, Trichrome stain, original magnification: 400×. **(H)** Cementoblasts are arranged in lines propagating perpendicular and in a radiating manner towards cementum and separated by inserting PDL fibers, Trichrome stain, 400×.

Regenerative bone formation was seen in most of the defect regions examined in this group with some areas devoid of bone and seemed to have been previously filled by β-TCP (Fig. 2A,B). Also, newly formed fibers of PDL were seen spanning the distances between the formed bone and the adjacent roots. These fibers exhibited irregular course patches of homogenous fiber aggregates and many blood vessels (Fig. 2C).

In addition, networks of fibrous tissues appeared to spread between the forming trabeculae and even connect them. Bone remodeling features were seen in both formed trabeculae which contained areas of the woven bone and in the bone border adjacent to the regenerating PDL (Fig. 2D,E).

In trichrome-stained sections, β-TCP particles were traced in the forming bone and surrounded by blood vessels. Interestingly bone formation was seen in rings at the outer periphery of these aggregates and even extending towards their center (Fig. 2F).

The organization and functional activity of osteoblasts and cementoblasts were examined in many sections of the specimens of this group. These cells reflected an advanced stage of regeneration. Voluminous osteoblasts appeared at the border of the forming bone between the inserting Sharpey’s fibers of the PDL (Fig. 2G). At the cementum side, inserting fibers of the PDL were seen accommodating between them cementoblasts arranged in streaming lines towards the cementum (Fig. 2H).

2 Control group

**Figure 3:**
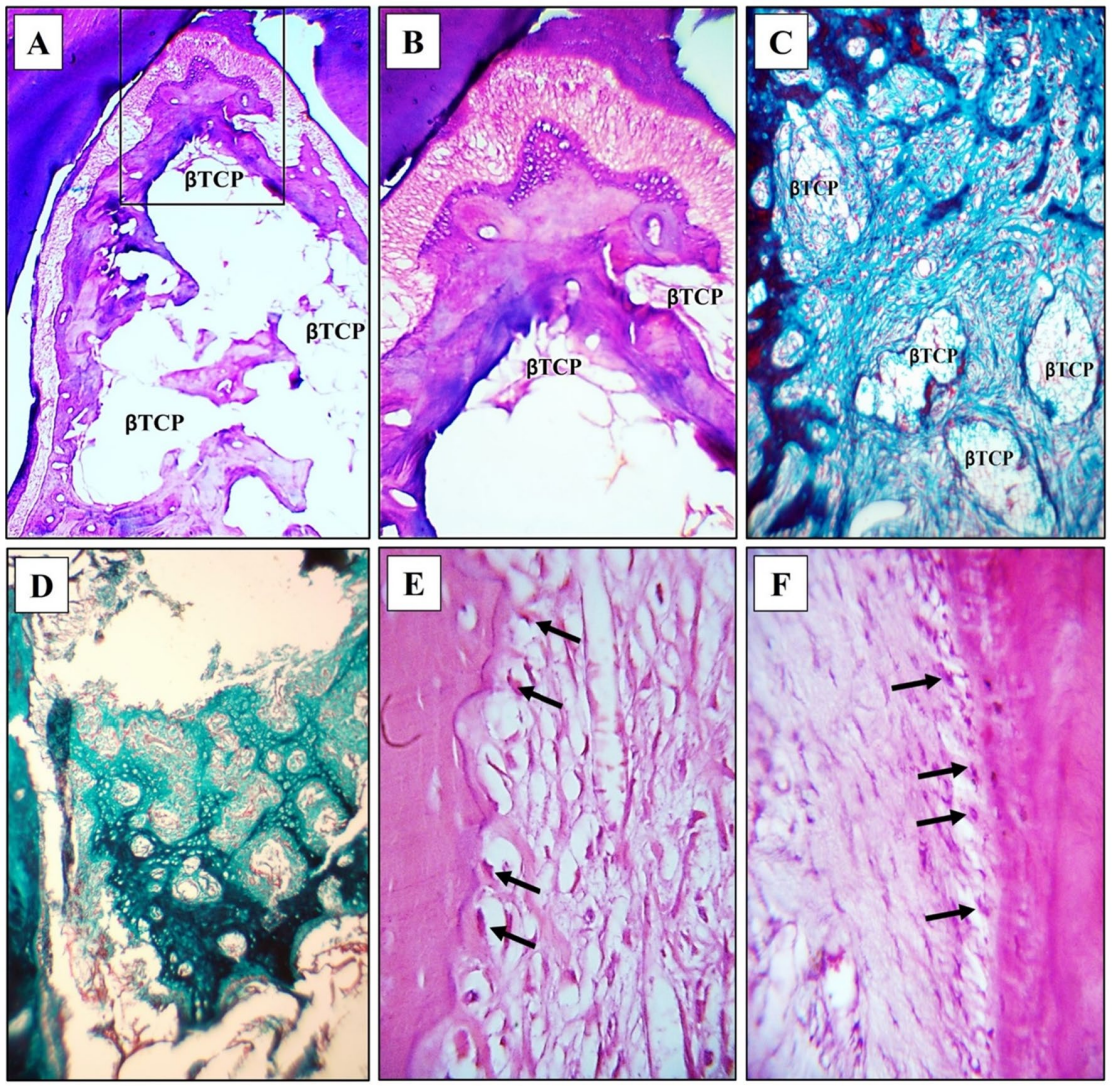
Control group at 4 weeks observational period **(A)** Formation of a limited amount of regenerative bone in one of the defects with β-TCP particles in the center. Forming PDL fibers are seen between the forming bone and the roots. H&E, 40×. **(B)** Higher magnification of the boxed area in the previous figure reveals the immature rims of bone and the forming PDL fibers, H&E, 100×. **(C)** Dense fibrous tissue formation in some parts of the defects with β-TCP particles among them. Note the very thin lines of bone formation at their edges, Trichrome stain, 40×. **(D)** Formation of immature and the adjacent highly cellular background, Trichrome stain, 400×. **(E)** Irregularly organized osteoblasts on the bone border (arrows) and facing disorganized PDL fibers and fibroblasts. H&E stain, 400×. **(F)** Flattened cementoblasts (arrows) adjacent to the newly forming cementum and facing disorganized PDL fibers and fibroblasts, H&E stain, 400×**.**

The formed bone in this group occupied the coronal parts and defect sides adjacent to mesial and distal roots. The central regions contained few amounts of forming bone surrounded by different figures of β-TCP which were greater than those seen in the experimental group (Fig. 3A). The formed bone at the most coronal regions of the defect consisted partly of mature and partly of immature bone with large osteocytes contained in partially vacated lacunae (Fig. 3B).

Regions lacking bone formation contained dense fibrous tissue with aggregates of β-TCP having very thin rims of bone formation that were seen less than those observed in the experimental group, (Fig. 3C) or contained formed woven bone on a background of fibrous tissue (Fig. 3D). Irregularly organized osteoblasts were seen adjacent to the regenerating bone between the irregularly inserting fibers of the formed PDL (Fig. 3E). Similarly, irregularly organized flat cementoblasts were seen at the cementum border (Fig. 3F).

#### Second observation period (8 weeks)

The results of this observation period were characterized by a greater amount of formed bone in the defects A better organization of all the regenerating components of the periodontium, and decreased amount of β-TCP were observed in the defects.

A. Experimental group

**Figure 4:**
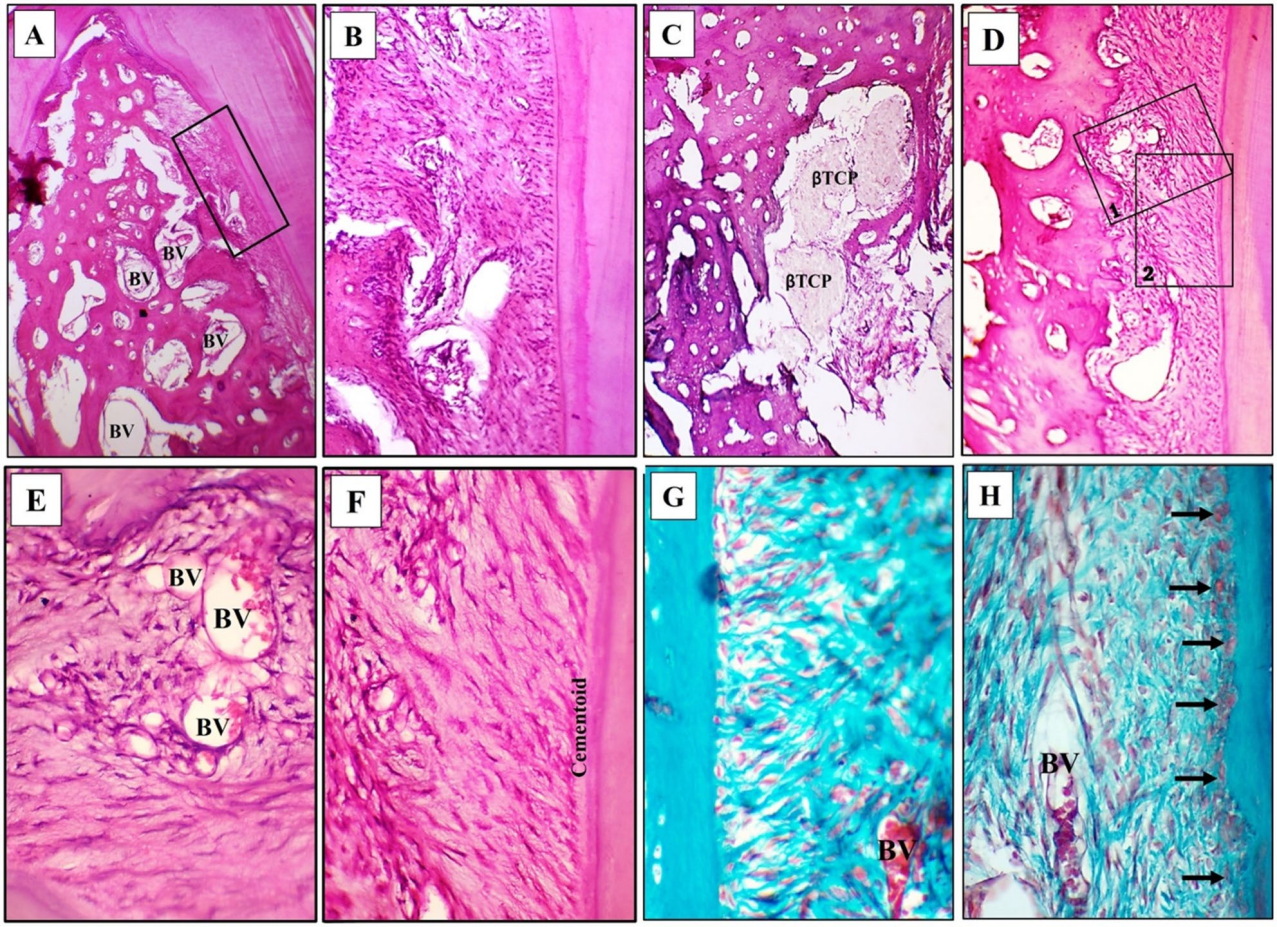
Experimental group at 8 weeks observational period **(A)** Prevalence of bone formation all over the defect. Intercommunicating thick trabeculae are seen enclosing rich blood supply (BV). Well-organized PDL structure is noted H&E, 40×. **(B)** The organized structure of the regenerating PDL and neighboring structures, H&E, 100X. (**C)** Denser bone formation in the defect center with enclosing remanent of the dissolving and restricted ß-TCP, H&E, 100×. (**D)** Longitudinal section of the regenerating periodontium in one of the defects. Well-organized PDL fibers with pronounced blood supply is seen. The alveolar bone border exhibits histological features of remodeling and regeneration, H&E, 100×. **(E)** Higher magnification of inset 1 in Fig. 4Grevealing the rich blood supply adjacent to the regenerating bone and the adjoining noticeable proliferation of osteoprogenitor cells and osteoblasts H&E, 400×. **(F)** Higher magnification of inset 2 in Fig. 4G revealing the fiber and cell density in the regenerating PDL with proper and typical fiber direction towards cementum. Cementoblasts are seen between the inserting fibers in close association with a layer of forming cementoid, H&E, 400×. **(G)** Insertion of the PDL fiber bundles in the regenerating alveolar bone while enclosing osteoblasts among them **(H)** Voluminous cementoblasts (arrows) resting on a layer of cementoid and accommodating the inserting Sharpey’ fibers. In the last 2 images note the cell density in the ligament and the blood supply (BV), Trichrome stain, 400×.

The amount of regenerating bone in this group appeared greater than that of the first observation period of the same group, with fewer figures of β-TCP among it. Thicker intercommunicating trabeculae were seen all over the defect and included considerable figures of blood supply (Fig. 4A,C).

Also, a more organized appearance of PDL structure prevailed; uniform thickness and fiber orientation could be followed from the regenerating bone towards the forming cementum. The PDL cell density among the fiber streams was a pronounced feature in this group (Fig. 4B,D,F).

The alveolar bone outline exhibited irregularities its outline facing the PDL indicating active and continuous remodeling. Rich blood supply was noticed adjacent to the regenerating bone which showed the proliferation of osteoprogenitor cells and osteoblasts (Fig. 4D,E).

Trichrome stain provided an excellent fiber stain for the observation of the PDL fiber and its insertion into the regenerating bone and cementum and for revealing the enhanced cellular activity in this group (Fig. 4G,H).

2 Control group

**Figure 5:**
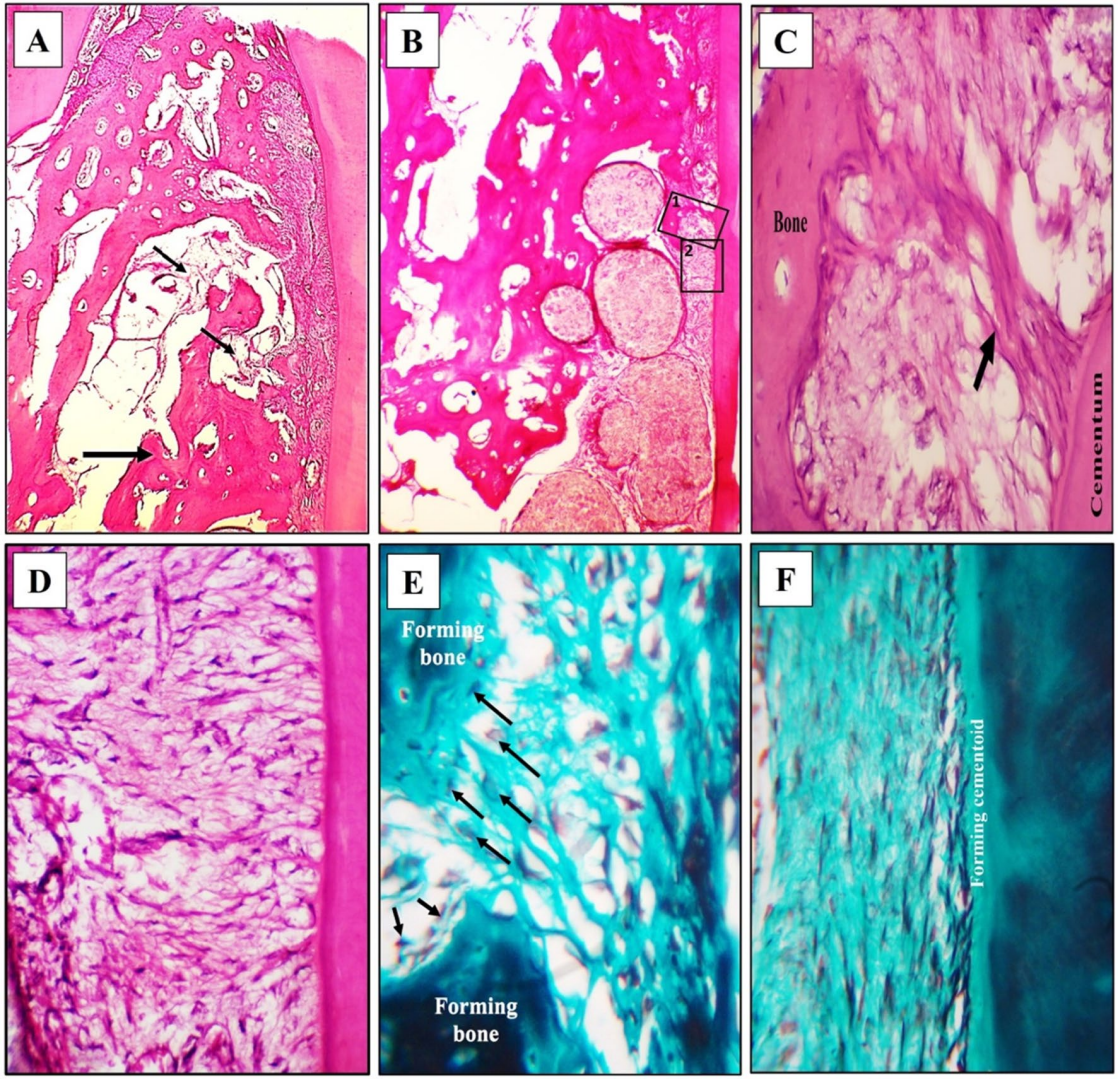
Control group at 8 weeks observational period **(A)** Prevalence of bone formation at the sides and coronal parts of the defect. Some remanent of β-TCP particles is seen in the areas devoid of bone formation (thin arrows). PDL fibers are seen along one side of the regenerating defect, H&E, 40×. **(B)** Part of the regenerating defect just apical to the level of the thick arrow in the previous figure revealing part of the formed bone and aggregates of β-TCP between it and the PDL, H&E, original magnification 100×. **(C)** Disorganized PDL fibers and cells with the appearance of a fiber bundle exhibiting disorganized thickness and course towards the forming bone (arrow), H&E, 400×. **(D)** interrupted thin PDL fibers and disorganized orientation of the fibroblast cells, Note the inconsistent fiber insertion in cementum H&E, 400×. (**E)** Irregularly organized fibers and osteoblasts at the border of the formed bone and between the inserting fibers (arrows), Trichrome stain, 400×. **(F)** Irregularly organized cementoblasts adjacent to cementum, Trichrome stain, 400×.

The amount of formed bone in the defect was not comparable to that of the experimental group of the same observational period. Some areas of the defect center were devoid of bone formation. However, the regenerative features in the defects were better than that of the same groups of the first observation period (Fig. 5A).

At the PDL side of some defects, large aggregates of β-TCP were seen intervening between the regenerating cementum and PDL and the already-formed bone. However, some of these masses exhibited frank dissolution of their content (Fig. 5B).

High magnification images of the PDL revealed the non-homogenous thickness of the fibers and random accommodation of the fibroblast cells, with some thick fiber bundles emerging from one zone on the root side and propagating in a disorganized manner towards the alveolar bone. Also, fiber insertion into the cementum was inconsistent (Fig. 5C,D).

In trichrome-stained sections, the cell size, fiber orientation, insertion, and cell organization in the central regions of the PDL as well as at the cementum side were revealed and emphasized the appearance in H and E-stained sections (Fig. 5E,F).

### Histomorphometry measurements

The histomorphometry assessment of the three measured parameters (Height of interradicular bone, thickness of bone trabeculae, and percentage of bone surface area) at the two observational periods has shown comparable results in both groups with greater values in the Aloe vera gel group (Table 1).

**Table 1:** Comparison between the experimental and control groups regarding the height of the newly formed bone, trabecular bone thickness, and the Percentage of the surface area of the newly formed bone after 4 and 8 weeks.

		Control group (n = 12)	Experimental group (n = 12)	Test (*P* value)
Height of the newly formed bone (mm)				
4 weeks	Mean (SD)	2.50 (0.16)	2.88 (0.23)	4.356 (< 0.0001*)
	Min–Max	2.25–2.71	2.58–3.21	
8 weeks	Mean (SD)	2.94 (0.26)	3.53 (0.16)	6.214 (< 0.0001*)
	Min–Max	2.47–3.31	3.35–3.81	
Test (*P* value)		4.309 (0.002*)	6.994 (< 0.0001*)	
Trabecular bone thickness (mm)				
4 weeks	Mean (SD)	0.23 (0.06)	0.43 (0.08)	6.321 (< 0.0001*)
	Min–Max	0.18–0.36	0.30–0.54	
8 weeks	Mean (SD)	0.37 (0.06)	0.53 (0.10)	4.390 (< 0.0001*)
	Min–Max	0.28–0.46	0.41–0.69	
Test (*P* value)		4.171 (0.002*)	5.765 (< 0.0001*)	
Percentage of surface area of the newly formed bone (%)				
4 weeks	Mean (SD)	44.61 (7.87)	55.46 (8.58)	2.946 (0.009*)
	Min–Max	30.04–56.15	43.08–69.55	
8 weeks	Mean (SD)	64.49 (11.16)	74.66 (6.64)	2.477 (0.023*)
	Min–Max	49.60–79.30	68.03–84.99	
Test (*P* value)		5.532 (< 0.0001*)	6.994 (< 0.0001*)	

*Statistically significant difference at *p* value < 0.05.

Significant values are in [bold].

After 4 weeks, the mean [± SD] height of the newly formed bone in defects treated with Aloe vera gel was significantly higher than the control group (2.88 [± 0.23] mm vs 2.50 [± 0.16] mm, respectively; *p* < 0.0001). Similarly, after 8 weeks, the mean height [± SD] was (3.53 [± 0.16] mm and 2.94 [± 0.26] mm, *p* < 0.0001) for the experimental and control groups, respectively. The trabecular bone thickness of the experimental group showed significantly higher values after 4 and 8 weeks (0.43 [± 0.08] mm, and 0.53 [± 0.10] mm, respectively; *p* < 0.0001) as compared to the control (0.23 [± 0.06], and 0.37 [± 0.06] mm, respectively; *p* < 0.0001).

After 4 weeks, the mean percentage of the new bone surface area in the defects treated with Aloe vera gel was 55.46 [± 8.58] % which was significantly higher than that in the control group 44.6 [± 7.87] %; *p* = 0.009. Additionally, after 8 weeks, it was significantly higher in the experimental group than in the control (74.66 [± 6.64] %, vs 64.49 [± 11.16], respectively; *p* = 0.023). Intragroup comparisons revealed a significant increase in bone height, thickness, and surface area after 8 weeks for the Aloe vera gel and the control group, respectively (all *p* values < 0.05).

## Discussion

The current study was designed to test if Aloe vera could be used in treating critical-sized Grade II furcation defects as it can stimulate cell proliferation and osteogenesis by inducing extracellular matrix synthesis and bone mineralization. The regenerative effect of Aloe vera in furcation bony defects were investigated histologically and histomorphometrically in surgically created defects in dogs.

The claimed null hypothesis was rejected by the study’s findings, which showed that Aloe vera, when mixed with β-TCP, resulted in more bone formation and new PDL fibers compared to β-TCP alone. Moreover, the organization and functional activity of osteoblasts and cementoblasts reflected an advanced stage of regeneration in the experimental group. Furthermore, the histomorphometry analysis results revealed that the increase in height, thickness, and percentage of the surface area of the newly formed bone was significantly higher in the Aloe vera group compared to the control group after 4 and 8 weeks.

The surgically created defect is considered an acute experimental model and it is valid if critical dimensions are established to hinder the occurrence of spontaneous regeneration thus it is comparable to periodontitis-induced defects. Grade II furcation defects regeneration, although possible, but still considered an unpredictable procedure, especially in terms of complete bone fill therefore numerous treatment modalities have been tested to regenerate these defects, including augmenting the defects by bone graft alone, or with membrane and sometimes the bone graft is mixed with a bioactive material to accelerate bone formation^24^.

The effect of Aloe vera extracts as a bioactive molecule and scaffold for periodontal tissue regeneration was evaluated in some in vitro studies and proved to stimulate periodontal ligament cell proliferation, and accelerate bone formation through increasing alkaline phosphatase activity, bone morphogenetic protein 2, type I collagen and mineral deposition^14,15^. However, this is the first experimental study on larger-sized animals (dogs) to assess histologically the effect of Aloe vera on bone regeneration which will recommend its use in bone regeneration of osseous defects caused by periodontitis in order to promote and accelerate bone formation.

The increased bone formation in the experimental group compared to the control group as proved by histological histomorphometry analysis results could be due to the anti-inflammatory effect of Aloe vera that gives chance for accelerated and efficient bone repair^26–28^. It was reported that the anti-inflammatory effect of Aloe vera could be due to the immunomodulatory effect of its main components including Acemannan, acetylated polysaccharides mannose, and veracilglucanos B and C, which regulate the expression of inflammatory mediators such as interleukins 6 and 8^27^.

Moreover, it was reported that Aloe vera is partially responsible for tissue repair through the action of one of its components which is a mannose-6-phosphate polysaccharide, that can activate macrophages, increases cytokine release, and stimulates an increase in the replication of fibroblasts and so considered as an immunostimulant material^29^. In addition, some studies have reported the positive effect of Acemannan as the main polysaccharide in Aloe vera, on stimulating the regeneration of bone, cementum, and periodontal ligament through the expression of growth factors^12,30^.

The present findings were similar to a study that tested the effect of topical application of Aloe vera on bone healing by histological examination and immunohistochemical evaluation of BMP7 on femur bone defect in Swiss rats. It was reported that Aloe vera could function as a bioactive molecule that stimulates the proliferation and differentiation of osteoblasts with high expression of BMP7 thus inducing new bone formation^31^.

Guided tissue regeneration (GTR) using β-TCP and collagen membrane was chosen as the golden standard technique to manage furcation defects in both experimental and control groups. Several studies used β-TCP in the management of furcation defects and concluded that it resulted in new bone formation and periodontal regeneration^32–34^. However, the use of β-TCP may result in substantial clinical improvements such as a reduction in probing depth (PD) but does not seem to promote the regeneration of the periodontal tissues^35^. Thus, superior outcomes in the studied groups are only attributed to Aloe vera’s osteoinductive and regenerative effects rather than GTR.

The potential limitation of this study is that the effect of Aloe vera was assessed in iatrogenic periodontal defects which are not caused by periodontitis. However, this is the only evidence-based methodology used in most of the histological studies in the literature^18,21^. The problem with inducing furcation boney defect by periodontitis in animals is that we cannot predict the amount of bone destruction or the size of the defect that will result from the inflammatory process and so the defects cannot be standardized. Moreover, the resulting defect from induced periodontitis may be not wide enough to be treated by GTR. Furthermore, the long-term influence and behavior of Aloe vera and its effect on bone-specific proteins such as alkaline phosphatase are not elaborated. Therefore, more comprehensive clinical investigations are required to confirm the clinical applicability of Aloe vera in managing periodontal defects.

In the current study, Aloe vera induced new bone, PDL, and cementum formation. This process is potentially regulated by immunostimulant Aloe Vera polysaccharides thus it is a candidate biomolecule for periodontal regeneration that can be used in all GTR procedures. On the other hand, more studies exploring bone-specific proteins and cytokines such as alkaline phosphatase, osteonectin, TNF- and IL-1 over longer periods are highly recommended using the same animal model. This is thought to enable the researchers to understand the full impact of Aloe vera on bone and periodontal tissue regeneration.

## Conclusions

These results suggest that Aloe vera potentiates the effect of β-TCP in bone regeneration and can be considered a promising choice for the treatment of furcation defects in humans. Aloe vera can accelerate the regeneration of all periodontal tissues including bone, PDL, and cementum.

## Data Availability

The data sets used and/or analyzed during the current study are available from the corresponding author upon reasonable request.
